# Poor outcome of revised resurfacing hip arthroplasty: 397 cases from the Australian Joint Replacement Registry

**DOI:** 10.3109/17453671003667176

**Published:** 2010-03-31

**Authors:** Richard N de Steiger, Lisa N Miller, Gareth H Prosser, Stephen E Graves, David C Davidson, Tyman E Stanford

**Affiliations:** ^1^Australian Orthopaedic Association National Joint Replacement Registry; ^2^Data Management and Analysis Centre, University of Adelaide; ^3^Perth Orthopaedic Institute, Fremantle Hospital and University of Western Australia, FreemantleAustralia

## Abstract

**Background and purpose:**

Recent years have seen a rapid increase in the use of resurfacing hip arthroplasty despite the lack of literature on the long-term outcome. In particular, there is little evidence regarding the outcome of revisions of primary resurfacing. The purpose of this analysis was to examine the survivorship of primary resurfacing hip arthroplasties that have been revised.

**Patients and methods:**

Over 12,000 primary resurfacing hip arthroplasties were recorded by the Australian Orthopaedic Association National Joint Replacement Registry between September 1, 1999 and December 31, 2008. During this time, 397 revisions for reasons other than infection were reported for these primary resurfacings and classified as acetabular, femoral, or both acetabular and femoral revisions. The survivorship of the different types of revisions was estimated using the Kaplan-Meier method and compared using proportional hazard models. Additionally, the outcome of a femoral-only revision was compared to that of primary conventional total hip arthroplasty.

**Results:**

Acetabular-only revision had a high risk of re-revision compared to femoral-only and both acetabular and femoral revision (5-year cumulative per cent revision of 20%, 7%, and 5% respectively). Femoral-only revision had a risk of re-revision similar to that of revision of both the acetabular and femoral components. Femoral-only revision had over twice the risk of revision of primary conventional total hip arthroplasty.

**Interpretation:**

Revision of a primary resurfacing arthroplasty is associated with a major risk of re-revision. The best outcome is achieved when either the femoral-only or both the acetabular and femoral components are revised. Technically straightforward femoral-only revisions generally have a worse outcome than a primary conventional total hip arthroplasty.

## Introduction

In recent years, there has been a considerable increase in the use of resurfacing hip arthroplasty. The renewed interest in this procedure has in part been the result of recent improvements in metal technology. There have been numerous publications presenting the short- to medium-term results of modern hip resurfacing (Back et al. 2005, Treacy et al. 2005, Hing et al. 2007, Heilpern et al. 2008, Marulanda et al. 2008, McMinn et al. 2008, Steffen et al. 2008, O'Neil el al. 2009). Theoretical advantages of resurfacing have included a reduced dislocation rate, improved function, increased range of movement, low wear rates, bone retention in the proximal femur, and ease of future revision. In particular, the latter claim has not been based on any scientific evidence.

In Australia, the use of modern resurfacing hip prostheses increased rapidly between 1999 and 2006. Resurfacing hip replacement accounts for 8% of all primary total hip arthroplasties recorded by the Australian Orthopaedic Association National Joint Replacement Registry (AOANJRR) up to the end of 2008. The registry has data on over 12,000 primary resurfacing hip arthroplasties and has previously reported on their outcome (AOANJRR Annual Report 2009). Registry data cannot help to determine some of the supposed benefits of resurfacing arthroplasty but they can help to determine the fate of further revision. We investigated the outcome of primary resurfacing hip arthroplasties that had been revised.

## Patients and methods

All hospitals undertaking arthroplasty in Australia provide data using special forms that are returned to the AOANJRR each month. Validation of registry data is undertaken by a sequential multi-level matching process against independently collected health department data. This process enables almost 100% data collection.

Of the 12,093 primary resurfacing hip arthroplasties reported to the registry between September 1, 1999 and December 31, 2008, there were 437 revisions recorded during that time. Of these revisions, 39 (9%) had been undertaken for infection. This analysis excludes these procedures for infection, and also 1 other procedure in which the surface replacement was removed for a diagnosis of metal sensitivity and no further prosthesis was re-inserted. We determined the outcome of the remaining 397 revision procedures that were undertaken for reasons other than infection.

The registry classifies revisions as major or minor. A major revision involves the exchange of a component that interfaces with bone and a minor revision involves all other types of revision. The major revisions are subdivided further into partial (acetabular or femoral) or total (acetabular and femoral) revisions. Unlike revisions of conventional total hip arthroplasty, where liner or femoral head changes can be performed, all revisions of primary resurfacing arthroplasty are major. The revisions of primary resurfacing arthroplasty were categorized as acetabular-only, femoral-only, or both acetabular and femoral, and their outcome compared.

Femoral-only revisions of primary resurfacing hip arthroplasty were also compared to the outcome of 141,611 primary conventional total hip arthroplasties recorded by the registry over the same time period (excluding primary diagnosis of fractured neck of femur and revision for infection). Acetabular-only revision and revision of both femoral and acetabular components were excluded from this comparison as an acetabular-only revision would leave the patient still with a resurfacing prosthesis, and revision of both components is clearly a major revision and as such is unlikely to have an outcome comparable to a primary conventional total hip arthroplasty.

### Statistics

Survivorship was estimated using the Kaplan-Meier method, with 95% confidence intervals. Age- and sex-adjusted hazard ratios (HR) from Cox proportional hazards models were used to compare the survivorship over the entire period. For each model, the assumption of proportional hazards was checked analytically by testing the significance of the interaction between the predictor and the log of time in the standard Cox model. The assumption of proportionality was not violated, and the hazard ratios presented are over the entire follow-up period. All tests were two-tailed at the 5% level of significance. Analysis was performed using SAS software version 9.2.

## Results

The registry has recorded the use of 13 different resurfacing prostheses. The Birmingham hip was the most frequently used, accounting for 70% of all primary resurfacing procedures. The ASR, Durom, and Mitch were the next most used prostheses (Table 1).

**Table 1.** Revision rates for primary resurfacing hip arthroplasty

Head component	Acetabular component	No. revised	Total no.	Obs. years	Revisions per 100 obs. years (95% CI)
ASR	ASR	64	1,073	2,814	2.3 (1.8–2.9)
Adept	Adept	4	292	525	0.8 (0.2–2.0)
BHR	BHR	269	8,427	34,340	0.8 (0.7–0.9)
Bionik	Bionik	5	119	181	2.8 (0.9–6.5)
Conserve	Conserve Plus	0	10	25	0.0 (0.0–15)
Conserve Plus	Conserve Plus	5	62	249	2.0 (0.7–4.7)
Cormet	Cormet	14	192	915	1.5 (0.8–2.6)
Cormet 2000 HAP	Cormet	10	95	460	2.2 (1.0–4.0)
Cormet HAP BiCoat	Cormet	10	287	534	1.9 (0.9–3.4)
Durom	Durom	37	767	2,223	1.7 (1.2–2.3)
Icon	Icon	2	96	196	1.0 (0.1–3.7)
Mitch TRH	Mitch TRH	7	534	627	1.1 (0.5–2.3)
Recap	Recap	8	137	255	3.1 (1.4–6.2)
Total		435	12,091	43,344	1.0 (0.9–1.1)
Note: 2 resurfacing hip procedures using only a Conserve resurfacing head and no acetabular component have not been included in this table.					

After excluding infection, the major reason for revision of primary resurfacing hip arthroplasty was fracture of the femoral neck (43%), followed by loosening/lysis (32%), metal sensitivity (7%), and pain (6%) (Table 2). The most common type of revision was a femoral-only revision (62%), followed by acetabular and femoral (29%), and acetabular-only (9%) (Table 3).

**Table 2.** Revision diagnosis for primary resurfacing hip arthroplasty by type of revision (excluding infection from revision of primary)

Revision diagnosis	Acetabular only	Femoral only		Acetabular and femoral		Total	
	n	n	%	n	%	n	%
Fracture	–	164	66	8	7	172	43
Loosening/lysis	25	52	21	51	45	128	32
Metal sensitivity	1	–	–	27	24	28	7
Pain	1	13	5	9	8	23	6
Avascular necrosis	–	10	4	5	4	15	4
Dislocation of prosthesis	2	4	2	8	7	14	4
Other	7	4	2	6	5	17	4
Total	36	247	100	114	100	397	100

**Table 3.** Revision rates for revised primary resurfacing hip arthroplasty, by type of revision (excluding infection from revision of primary)

Revised primary resurfacing hip	No. revised	Total no.	Obs. years	Revisions per 100 obs. years (95% CI)
Acetabular only	6	36	123	5.0 (1.8–11)
Femoral only	13	247	735	1.8 (0.9–3.0)
Acetabular and femoral	5	114	219	2.3 (0.7–5.0)
Total	24	397	1078	2.2 (1.4–3.3)

Of the 397 revisions of primary resurfacing hip arthroplasty undertaken for reasons other than infection, 24 underwent a further revision. The reasons for re-revision included loosening/lysis (n = 9), infection (n = 6), and dislocation (n = 4). The most common type of re-revision was femoral-only revision, followed by acetabular and femoral, and acetabular-only (Table 4).

**Table 4.** Re-revision diagnosis for revised primary resurfacing hip arthroplasty, by type of revision (excluding infection from revision of primary)

Re-revision diagnosis	Acetabular only (36)	Femoral only (247)	Acetabular and femoral (114)	Total (397)
	n	n	n	n
Loosening/lysis	3	6	–	9
Infection	1	4	1	6
Dislocation of prosthesis	2	1	1	4
Fracture	–	2	1	3
Other	–	–	2	2
Total	6	13	5	24
Note: numbers in parentheses refer to the number of revisions of the primary resurfacing procedure for that type of revision.				

**Figure 1.**
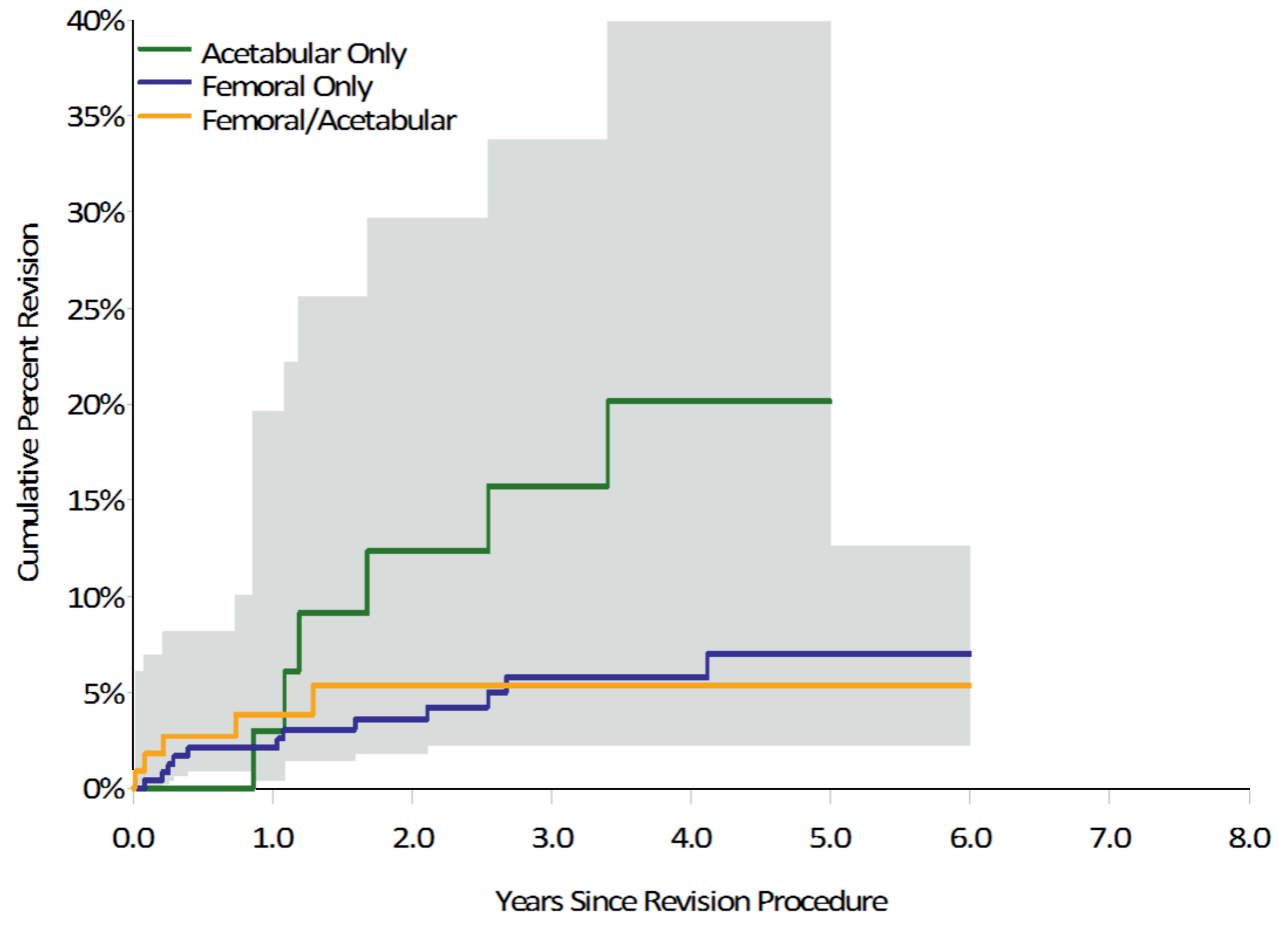
Cumulative percent revision of revised primary resurfacing hip arthroplasty, by type of revision (excluding infection from revision of primary).

The subsequent cumulative per cent revision of the 397 revisions of primary resurfacing hip arthroplasty was 9% (6–13) at 5 years. This varied depending on the type of revision, with acetabular-only revision having the highest risk of re-revision. Acetabular-only revision had a higher risk of re-revision than femoral only revision (HR = 3 (1.2–10), p = 0.02); however, there was no statistically significant difference when compared to acetabular and femoral revision (HR = 3 (0.9–9), p = 0.09). The risk of re-revision was similar between femoral-only and acetabular and femoral revision (HR = 0.8 (0.3–3), p = 0.7). The 5-year cumulative per cent revision was 20% (10–40) for acetabular-only revision, 7 (4–13) for femoral-only revision, and 5 (2–13) for revision of both components (Figure 1). For the acetabular-only revisions, 3 patients had further revision for loosening/lysis, 2 for dislocation, and 1 for infection.

**Figure 2.**
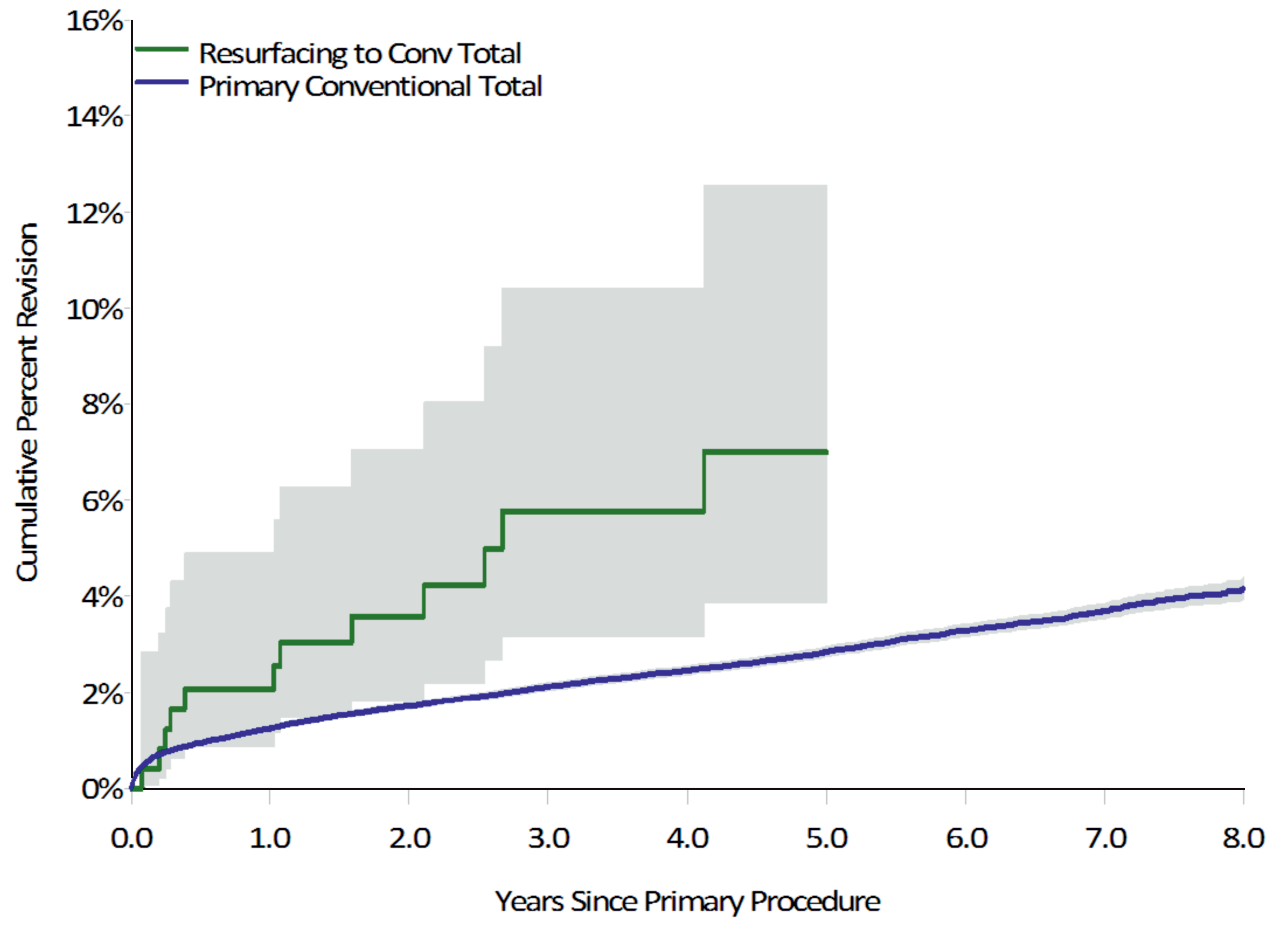
Cumulative percent revision of primary conventional total hip arthroplasty and femoral-only revision of primary resurfacing hip arthroplasty (excluding primary diagnosis of fractured neck of femur and also excluding infection from revision of primary).

A femoral-only revision of a primary resurfacing hip arthroplasty converts the joint replacement to a conventional total hip. However, the rate of revision was higher when comparing the outcome of femoral-only revision to the outcome of primary conventional total hip arthroplasty (HR = 2 (1.4–4), p = 0.001) (Figure 2). At 3 years, the cumulative per cent revision of femoral-only revision of primary resurfacing was 7% (4–13) as compared to 2.8% (2.7–3.0) for primary conventional total hip arthroplasty.

## Discussion

There have been few reports regarding the outcome of revised primary resurfacing arthroplasty. Grammatopoulos et al. (2009) identified 53 hips that had undergone metal-on-metal hip resurfacing and required a revision after an average of 1.6 years. Of these, 16 were revised for pseudotumors, 21 for fracture, and 16 for other reasons. The percentage of fractures (39%) as a cause of revision of the primary was similar in our analysis (43%). The Australian Joint Replacement Registry does not specifically identify pseudotumor as a diagnosis, but uses the diagnosis of metal sensitivity to cover all cases of metallosis. We suspect that metal sensitivity is under-diagnosed and that a proportion of revisions for early loosening/lysis may be the result of metal sensitivity.

A limitation of the Grammatopoulos study is that the pool of primary procedures that the revisions were derived from has not been qualified. As a consequence, it is not possible to determine whether the proportion of revision diagnoses reported in that study are representative of all revisions from that population of primary resurfacing procedures. The conclusion of the study was that the diagnosis of pseudotumor had a higher risk of re-revision than revisions for fracture or other causes. Of the 16 hips diagnosed with pseudotumor, 5 required further revision with 1 awaiting surgery. This conclusion may be correct; however, the validity remains uncertain as it is not clear whether this is a selective group of revisions. What does appear apparent is that the risk of re-revision following a revision of a primary resurfacing procedure is high in at least one subpopulation of these revision procedures.

To properly investigate the outcome of revision, the analysis must take into account the full chronological history from the primary to the second revision procedure. To achieve this requires a full understanding of the complete primary population and complete capture of all revisions from that population. It is clear that a large number of primary procedures with a long follow-up is required.

Ollivere et al. (2009) reported on the early failure of a consecutive series of 493 Birmingham hip resurfacings in a 2-center, 5-surgeon series. The authors stated that this series is the largest independent report of a single-implant metal-on-metal resurfacing (to date). Of these, 13 (3%) were revised—9 of which had macroscopic and histological evidence of metallosis. All patients were revised to a cemented Exeter stem with a variety of bearing surfaces, with no mention of whether or not the cup was retained. There was no information provided on the outcome of these revisions. While these authors were able to report on 13 revisions of the primary implant, their study emphazes the need for a much larger series to analyze the outcome of the first revision.

Our analysis is based on 397 revisions from over 12,000 primary resurfacing procedures. We believe that it is unlikely that full chronological data on the outcome of revisions of resurfacing procedures could be obtained from any source other than a registry. The 5-year cumulative percent revision of revised primary resurfacing procedures was 9% and this varied depending on the type of revision. Our analysis has shown that acetabular-only revision is associated with the highest risk of re-revision and that femoral-only revision has the same risk of re-revision as revising both the acetabular and femoral component.

One of the theoretical advantages of a resurfacing procedure is that retention of the proximal femur enables ease of revision. Although this may be technically correct, the finding that femoral-only revision has the same risk of re-revision as revising both components would suggest that the ease of revision cannot be extrapolated into a better outcome. Furthermore, conversion of a primary resurfacing to a conventional total by a femoral-only revision has more than twice the risk of revision than a primary conventional total hip arthroplasty.

Another finding of our analysis was that although infection is an uncommon cause of revision of a primary resurfacing arthroplasty (0.3%), it is a major cause of re-revision and accounted for 6 of the 24 re-revision procedures. These revisions have a shorter follow-up period and yet a substantial increase in the risk of infection compared to primary resurfacing.

In summary, the most common reason for revision of a primary resurfacing hip arthroplasty in the first 8 years of follow-up is fracture. Revision following a femoral fracture usually involves replacement of the femoral prosthesis only. Although technically straightforward, femoral-only revision has a higher risk of revision than primary conventional total hip arthroplasty. This early risk of re-revision is similar to revision of primary resurfacing arthroplasty that requires replacement of both the acetabular and femoral components. If there is a need to revise a primary resurfacing, the best outcome is achieved by revising either the femoral-only or both acetabular and femoral components.
